# Unsupervised modeling of mutational landscapes of adeno-associated viruses viability

**DOI:** 10.1186/s12859-024-05823-5

**Published:** 2024-07-02

**Authors:** Matteo De Leonardis, Jorge Fernandez-de-Cossio-Diaz, Guido Uguzzoni, Andrea Pagnani

**Affiliations:** 1 DISAT, Politecnico di Torino, Corso Duca degli Abruzzi, 10129 Torino, Italy; 2 Laboratoire de Physique de l’Ecole Normale Supérieure, CNRS, PSL University, Sorbonne Université, Universite, Paris-Cité, 75005 Paris, France; 3 Italian Institute for Genomic Medicine, IRCCS Candiolo, SP-142, 10060 Candiolo, Italy

**Keywords:** Computational biology, Directed evolution, Machine learning, Deep mutational scanning

## Abstract

Adeno-associated viruses 2 (AAV2) are minute viruses renowned for their capacity to infect human cells and akin organisms. They have recently emerged as prominent candidates in the field of gene therapy, primarily attributed to their inherent non-pathogenic nature in humans and the safety associated with their manipulation. The efficacy of AAV2 as gene therapy vectors hinges on their ability to infiltrate host cells, a phenomenon reliant on their competence to construct a capsid capable of breaching the nucleus of the target cell. To enhance their infection potential, researchers have extensively scrutinized various combinatorial libraries by introducing mutations into the capsid, aiming to boost their effectiveness. The emergence of high-throughput experimental techniques, like deep mutational scanning (DMS), has made it feasible to experimentally assess the fitness of these libraries for their intended purpose. Notably, machine learning is starting to demonstrate its potential in addressing predictions within the mutational landscape from sequence data. In this context, we introduce a biophysically-inspired model designed to predict the viability of genetic variants in DMS experiments. This model is tailored to a specific segment of the CAP region within AAV2’s capsid protein. To evaluate its effectiveness, we conduct model training with diverse datasets, each tailored to explore different aspects of the mutational landscape influenced by the selection process. Our assessment of the biophysical model centers on two primary objectives: (i) providing quantitative forecasts for the log-selectivity of variants and (ii) deploying it as a binary classifier to categorize sequences into viable and non-viable classes.

## Introduction

Recently, there has been a burgeoning interest in combining high-throughput sequencing with machine learning techniques to extend predictions beyond the boundaries of experimentally observed sequences [1]. While previous studies predominantly concentrated on a single protein property directly associated with the selection criteria, such as binding, stability, or catalytic activity [2], a few investigations have showcased the feasibility of inferring multiple physical properties, including those that are not directly measurable [3]. Notable examples of success include predicting thermal stability based on binding affinity measurements [4, 5] and deducing specificity profiles of transcription factors from the selective enrichment of DNA sequences [6, 7]. The key to these achievements lies in the application of biophysics-inspired models, which incorporate thermodynamic principles to yield interpretable predictions.

The combination of high-throughput *in-vitro* selection methods with high-throughput sequencing has been key to identifying protein variants of a given wild-type sequence with target functional activity [8–10]. However, it’s crucial to recognize that all experimental approaches are constrained by the maximum library size, typically falling within the range of $10^{8}$ (for yeast display), $10^{10}$ (for phage display), to $10^{15}$ (for ribosome display). Although these numbers may seem substantial, they represent only a minuscule fraction of the vast sequence space (for example, there are $20^{28}\sim 2\times 10^{36}$ possible protein sequences of length 28). Lately, there has been a significant surge of interest in directed evolution experiments (both in vitro and in vivo) aimed at engineering the capsid of adeno-associated virus (AAV) [11–14]. AAV2 are small non-enveloped viruses of a typical dimension of about 20 nm. They are provided with a spherical capsid composed of a mixture of three proteins (VP1, VP2, and VP3) giving structure to 60 subunits arranged with icosahedral symmetry. While they can infect humans and other primate species, there are currently no known diseases caused by them. This, besides other technical motivations, makes them ideal candidates as viral vectors in gene therapy as shown in several clinical tests.

The use of AAV2s in gene therapy is conditioned by their potential to infect cells which in turn depends on their capacity to assemble a viable capsid, meaning that they assemble an integral capsid that packages the genome. From the mechanical point of view, much interest has been attracted by the idea of understanding the assembly process of the viral capsid [15], bringing considerable insights about the patterns that lead the capsid formation starting from its basic building blocks (individual capsid proteins, oligomers or capsomers). The capsid formation can be described as a nucleation process driven by three free-energy contributions: (i) the energy gain in growing a partial shell given by the binding energy between capsid proteins, (ii) a domain wall energy penalty due to missing contacts at the border of the shell, and (iii) the elastic energy due to the curvature of the shell shape. Thanks to experimental observations and simulation analysis, it is now possible to highlight the leading factors that make the assembly possible in different mechanical conditions and also the patterns through which the capsid can assume the correct shape. A bottleneck in achieving the desired phenotypic traits of the AAV genotype is associated with capsid production, where the majority of sequence variants fail to either assemble or package their genome. This phenomenon is commonly referred to as *viability*.

Here, we address the problem of forming a viable capsid from a deep mutational scanning (DMS) experiment perspective. In particular, we study how mutations of a particular section of the CAP region affect capsid formation. We stress that the assembly of a stable capsid and the packaging of the genome are two concurrent processes necessary for the functionality of the viral vector. Unfortunately, the experimental strategy used in [16] does not permit to discriminate assembly from packaging effects. As the mutated part of the capsid encompasses buried, surface, and interface regions, it is not likely to be involved in the packaging process. We argue that residues involved in packaging are more likely to be located in the interior of the capsid shell. In any case, given the partial understanding of these complicated mechanisms, we cannot exclude that variants might impact some determinants of genome packaging without altering the capsid formation. Following this reasoning to the extreme, in principle, an empty capsid could form without being detected by sequencing. This is a limitation of the chosen experimental technique, and one must be aware that the viability signal might be affected by packaging-related effects. The idea of this work is to devise a new machine-learning strategy that improves currently employed computational techniques to analyze data from DMS experiments. Instead of training a deep neural network to solve a regression problem where the sequences are the input of the model and the output is the selectivity of the sequence, we aim to develop a biophysical model of a general DMS experiment, with physically interpretable parameters that remain comparable between different experimental realizations. On top of this advantage, we want to show how a biophysical model can lead to a more robust inference: less biased by experimental noise and more accurate in predicting the selectivity of the variants especially when we are interested in comparing it between different variants. We remark that our approach, even if inspired by a biophysical model, aims at inferring a statistical proxy that is not exactly the stability energy of the capsid that can be measured or estimated from dynamical simulation. Even if these quantities can be correlated, this should be checked a-posteriori when data on capsid stability are available.

For this purpose, we analyzed data collected in a massive study on capsid diversity ( [16]). In this study a DMS experiment has been performed testing a large library of viruses with diverse capsid composition, these data have been then used to train different machine learning models to infer the mutational landscape for capsid viability and use it to generate viable capsids as different as possible from the WT one. The statistical model employed in our approach is an energy-based model, expressed through a combination of convolutional layers (accounting for variations in sequence lengths within the training sets) and a dense layer, responsible for mapping sequences to energy levels. Notably, the performance of this biophysics-inspired model proves intriguing even in a more straightforward classification scenario, where it is applied to the binary task of distinguishing viable from non-viable sequences.

## Experiment and data

The data used in our analysis are derived from a recent comprehensive study [16]. In this work, the variability of protein capsids in AAV2 and their ability to remain viable for DNA packaging was investigated by developing a deep learning method to estimate the mutational landscape for capsid viability and generate new diverse viable capsids that differ significantly from those found in nature. The analysis was focused on a specific region comprising 28 amino acids near the 3-fold symmetry axis of the icosahedral AAV2 VP1 protein. This region is known to play a crucial role in viral production. The phenotypic trait selected for in the experiment is the virus viability and it was quantitatively assessed through high-throughput screening experiments to collect data on a wide range of variants. To do so, a series of libraries of designed capsid proteins were synthesized and inserted into the virus genome’s cap region. Plasmids encoding various capsid structures were cloned to create an extensive plasmid library. These plasmids were first sequenced and then transfected into cells. After viral production, the DNA was extracted and sequenced once again, resulting in two sets of reads for each tested variant. These reads provided information about the abundance of each sequence before and after transfection.

The training set for our algorithm comprises three data sets that differ considerably one from another in both size (number of unique variants) and diversity (distance from the wild-type sequence). To assess their diversity, we consider the Levenshtein distances (from now on we will always imply this particular metric whenever we talk about distance) of the sequences from the wild-type one and pairwise distances between different experiments. Starting from *experiment-1* which contains only the WT and single mutations, increasingly more diverse sequences are tested in the following experiments.

**Fig. 1:**
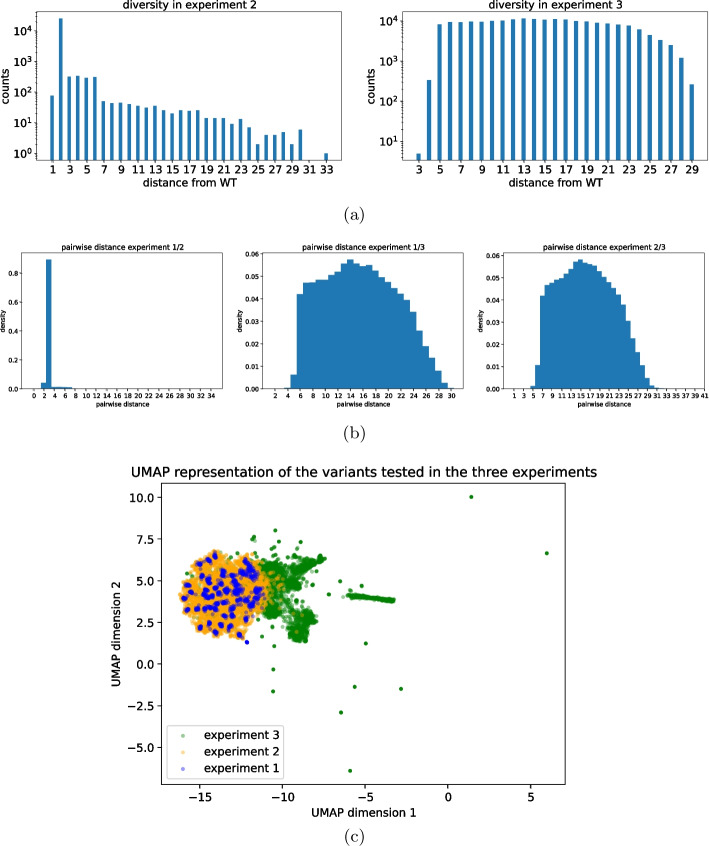
**a** Distances from the WT sequence of the tested sequences in each of the two experiments performed in [16]. **b** Distances between all pairs of sequences tested in different experiments. **c** UMAP projection of the sequences tested in the three experiments. Due to the high number of variants tested in *experiment-2* and *experiment 3*, 5000 sequences have been randomly sampled from these experiments

Figure 1b shows the distribution of the distances between all pairs of sequences in the three different experiments. To have a global overview of variants experimentally tested, Fig. 1c shows a schematic of the sequences tested in all experiments, this has been realized using the UMAP algorithm representation for dimensionality reduction [17]. Table 1, instead, summarizes the main features of the three datasets.

**Table 1:** Relevant information about size, quality, and diversity of the dataset tested in each experiment

	Experiment-1	Experiment-2	Experiment-3
Unique variants	1085	26961	203635
Depth round 0	2423857	14353318	49376644
Depth round 1	3491860	23411926	194466876
Coverage round 0	2234	532	242
Coverage round 1	3218	868	955
Distance from WT round 0 ($\mu \pm \sigma$)	$0.9 \pm 0.2$	$3 \pm 2$	$13 \pm 5$
Distance from WT round 1 ($\mu \pm \sigma$)	$0.9 \pm 0.3$	$3 \pm 2$	$10 \pm 4$

## Methods

In this section, we describe the details of our biophysical-inspired statistical model, and describe the different training and test datasets used. The model defines a statistical *energy* that we consider as a proxy to the sequence viability. Strictly speaking, viability is a discrete phenotypic trait that in the series of experiments conducted that constitute our training set [16] is assessed in terms of sequencing counts. The boolean nature viable/non-viable is eventually induced with a statistical analysis *a-posteriori*. To compare the performance of the our model in solving the simpler boolean classification task, we also introduce a standard classifier with the same architecture as the energy-based model.

### Model

The machine-learning method we propose utilizes a statistical model that aims to capture the entire experimental pipeline conducted to assess the ability of variants to package the genome within the capsid. The structure of this model is very general and permits the analysis of any DMS (Deep Mutational Scanning) experiment. As previously discussed in [18], the model comprises three main steps: selection, amplification, and sequencing.

During the selection phase, mutated viruses are introduced into tissue cells, where their survival depends on their ability to form functional capsids. Typically, only a small fraction of the initial population of viruses is selected during this phase. Following selection, viral DNA is extracted from a sample of supernatant and undergoes amplification. Finally, the amplified DNA is sequenced to gather information about the genetic composition of the selected viruses.

#### Selection

We examine a population of AAV2s (adeno-associated viruses), each carrying a mutated sequence of the gene of interest. Let $N_s$ represent the count of viruses containing sequence *s* in the initial library. From a biophysical perspective, we assume that the functional characteristics of the viral variant are determined by the thermodynamic states adopted by the protein products resulting from the mutated gene. The expressed protein copies of variant *s* can either bind together, leading to the assembly of a functional capsid, or their structural properties may hinder capsid formation. The energies of these two states will be denoted by $E^{\text{capsid}}_s$ and $E^\mathrm {dissol.}_s$, respectively. Since survival depends on the ability to form the capsid, the probability of selection for viruses carrying variant *s* (or *selectivity*) can be computed according to the Boltzmann law

1 $$\begin{aligned} p_s&= \frac{\mathrm e^{\mu ^{\text{capsid}} - E^{\text{capsid}}_s}}{\mathrm e^{\mu ^{\text{capsid}} - E^{\text{capsid}}_s} + \mathrm e^{\mu ^\mathrm {dissol.} - E^\mathrm {dissol.}_s}} \nonumber \\&= \frac{1}{1 + \mathrm e^{\mu - E_s}} \end{aligned}$$

where $\mu ^{\text{capsid}}, \mu ^\mathrm {dissol.}$ are chemical potentials associated with each state, and we defined $\mu := \mu _\mathrm {dissol.} - \mu _{\text{capsid}}$ and $E_s:= E^\mathrm {dissol.}_s - E^{\text{capsid}}_s$. From now on, we define the pair $E_s, \mu$, energy specificity, and chemical potential respectively. With these definitions, $p_s$ assumes the well-known Fermi-Dirac distribution form for a two-level system. In the large $N_s$ limit, one can expect small fluctuations in terms of the number of selected sequences. Therefore, out of the $N_s$ viruses carrying sequence *s* in the initial library, we assume $N_s p_s$ are selected by their ability to form the capsid. This approximation is sometimes referred to as *deterministic binding approximation* [18].

#### Energy specificity

The capsid-formation energy $E_s$, depends on the ability of protein products to bind together and assemble the capsid. Protein binding depends on sequence *s*, and in full generality this dependence is complex. In analogy with the reference study, we use one of the neural network models tested in [16] to parametrize the energy mapping specificity. The network is composed of two convolutional layers and then three fully connected ones (see Sect. 3 in the Supplementary Material for the detailed structure).

We denote the set of parameters of this network (weights and biases) by $\Theta$. Having specified the network architecture, selectivities then become functions of $\Theta$ and the chemical potential,

2 $$\begin{aligned} p_s(\Theta , \mu ) = \frac{1}{1 + \mathrm e^{\mu - E_s(\Theta )}} \end{aligned}$$

Although other choices of energy function could be used in principle, the convolutional structure chosen here allows us to handle sequences of different lengths as explained in detail in Sect. 2 in the Supplementary Material.

#### Amplification

Empirically, it turns out that only a small fraction of viruses survive the selection phase. To replenish the original total population size, after the selection round an amplification phase through PCR is performed. We assume for simplicity that this process is uniform across all sequences. Then, denoting by $N_s'$ the abundance of sequence *s* after amplification,

3 $$\begin{aligned} N_s'\propto \frac{p_s N_s}{\sum _\sigma p_\sigma N_\sigma } \end{aligned}$$

#### Sequencing

In the experimental pipeline of [16], sequencing is performed at two different steps of the experiment: (i) when the initial combinatorial library is inserted into the plasmids ( but prior to transfection into the target cells), and (ii) after the viral extraction from the cell. It is clear that the target phenotype observed is the *viral viability*, i.e. the ability of the variant to remain active and capable of forming a stable capsid.

If we assume that each variant is sequenced with a probability proportional to its abundance, the probability of observing a count $R_s$ for variant *s* conditional to the (unobservable) number of variants $N_s$, follows the multinomial distribution:

4 $$\begin{aligned} {\mathcal {P}}({\textbf{R}}|{\textbf{N}}) = \frac{(\sum _s R_s)!}{\prod _s(R_s!)}\frac{\prod _s N_s^{R_s}}{\left( \sum _s N_s\right) ^{\sum _s R_s}} \end{aligned}$$

Here and in the following we indicate with ${\textbf{R}}$, ${\textbf{N}}$, and ${\textbf{p}}$ the vectors of read counts $R_s$, variant abundances $N_s$, and selectivities $p_s$, respectively. Similarly, for the sample taken after selection and amplification,

5 $$\begin{aligned} {\mathcal {P}}({\textbf{R}}'|{\textbf{N}}, {\textbf{p}})&=\frac{(\sum _s R_s')!}{\prod _s(R_s'!)}\frac{\prod _s N_s'^{R_s'}}{\left( \sum _s N_s'\right) ^{\sum _s R_s'}} \end{aligned}$$

6 $$\begin{aligned}&=\frac{(\sum _s R_s')!}{\prod _s(R_s'!)}\frac{\prod _s(p_s N_s)^{R_s'}}{\left( \sum _s p_s N_s\right) ^{\sum _s R_s'}} \end{aligned}$$

Note that ${\mathcal {P}}({\textbf{R}}'|{\textbf{N}}, {\textbf{p}})$ depends on the selectivities $p_s$, which in turn depend on the parameters $\Theta ,\mu$, see (2).

### Maximum likelihood inference

The model defined so far has several parameters that must be inferred from data: the parameters of the neural networks specifying the mapping from sequence to energy $E_s$, that we hereby denote by $\Theta$, the chemical potentials $\mu _s$, and the variant abundances in the initial library $N_s$. As a function of these parameters, the model log-likelihood can be written:

7 $$\begin{aligned} \begin{aligned} {\mathcal {L}}(\Theta , \varvec{\mu }, {\textbf{N}})&= \ln {\mathcal {P}}({\textbf{R}}|{\textbf{N}}) + \ln {\mathcal {P}}({\textbf{R}}'|{\textbf{N}}, {\textbf{p}}) \\&= \sum _s R_s\ln N_s + \sum _s R_s'\ln (p_s N_s) \\&\qquad - \left( \sum _s R_s\right) \ln \left( \sum _s N_s\right) - \left( \sum _s R_s'\right) \ln \left( \sum _s p_s N_s\right) + \text {const.} \end{aligned} \end{aligned}$$

Note that the dependence on the parameters $\Theta ,\mu$ arises from the dependence of the selectivities, (2). Following [19], we then learn $\Theta , \varvec{\mu }, {\textbf{N}}$ from the sequencing data by numerical maximization of the likelihood,

8 $$\begin{aligned} \max _{\Theta , \varvec{\mu }, {\textbf{N}}} {\mathcal {L}}(\Theta , \varvec{\mu }, {\textbf{N}}) \end{aligned}$$

See Sect. 4 in the Supplementary Material for details on the optimization procedure.

Once the optimal parameters have been obtained, the learning can be validated by assessing how the quantities observed experimentally correlate with the ones inferred. To be more precise, we look at the correlation between $p_s$ and the ratio $\theta _s = R_s'/R_s$, usually called *empirical selectivity* [20], on a subset of sequences that are not used during training of the model. Barring sampling noise, $\theta _s$ should be proportional to the selectivities $p_s$ inferred by the model. Unfortunately in this experiment, only one round of selection has been performed; therefore it is not possible to use information from multiple rounds to filter out experimental noise as described in [20] and already exploited in [18].

### Binarizing the output of the energy-based model

The most powerful feature of this machine learning method is that it is capable of inferring quantitatively the selectivity of every single variant. Anyway, there are cases in which the useful information is less specific and one can be only interested in knowing if a variant is able or not to perform its task. In these cases, one can use the model to perform a binary classification setting a threshold value for $p_s$ and classifying accordingly the variants.

**Fig. 2:**
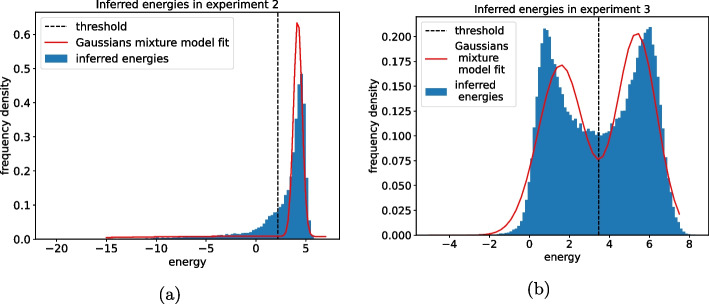
Fit performed on the histogram of inferred energies on the two test experiments. This provides an unsupervised estimate of the threshold for binary classification of the sequences as viable or non-viable. The energy distribution has been fitted with a bimodal Gaussians mixture distribution and the valley in between the two peaks has been chosen as threshold value

One unsophisticated but sensible way to set such a threshold is to fit a bimodal Gaussian distribution from the values of the inferred energies $E_s$. If the phenotypic trait has a binary nature and the test set is more or less balanced between viable and non-viable sequences that can be recognized by the model, the energy values will cluster around two values: a high energy value for non-viable variants and a low energy value for the viable ones. Thus, we decided to fit the distribution of the energy by two Gaussians mixture model. Of course, depending on how overlapping the two Gaussians turn out from the fit, different strategies can be used to define a threshold. In our case, as shown in Fig. 2, the two Gaussians are separated enough to produce a minimum in the mixture between the two peaks. We decided to use this value for the threshold as it is the most robust choice in the sense that a small perturbation around this value produces a minimal change in the classification. Of course, being our method entirely unsupervised, we have no unique way to set the threshold. The other option, as done for instance in [16], is to use a fully supervised strategy on variants already annotated as viable/non-viable.

As a semi-supervised check, one could only use the viable/non-viable annotation just to set the ”optimal threshold” after having inferred unsupervisedly the energy model. In Sect.  4 we will compare the discrepancy in classification when we use the threshold obtained as described here and the case in which we use the information on the viability of the test sequences to find an ”optimal threshold”; we do this to show that the threshold found by the naïve bimodal fit is already almost ”optimal”.

### Data binarization

To validate our model, we need to divide data into viable/non-viable classes. To do so, first an empirical proxy for the ”fitness” of the variants is computed, this quantity is usually referred to as *empirical (log)selectivity* and it is defined as the following

9 $$\begin{aligned} \theta _s = \log \frac{R'_s}{R_s} \end{aligned}$$

**Fig. 3:**
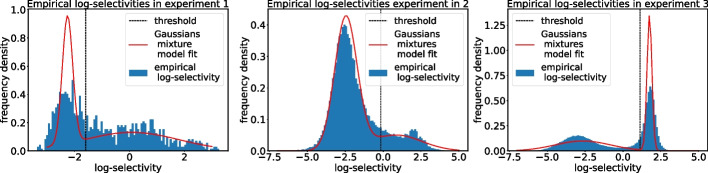
Fit performed on the histogram of empirical log-selectivities of each experiment to determine the threshold value for binary labeling of the sequences as viable or non-viable. The empirical distribution has been fitted with a bimodal Gaussians mixture distribution and the valley in between the two peaks has been chosen as threshold value

Then these quantities can be used to assign a label to each sequence. In [16] they have assigned a binary label to each variant setting a threshold for this quantity. The procedure we used to assign this threshold value is similar to the one used in the reference study. By direct inspection of the empirical log-selectivity distribution Fig. 3, one can see that they follow a bimodal distribution. We consider sequences near the rightmost peak as viable and the remaining as non-viable. In analogy with what we have done for the model energy, we fitted a bimodal Gaussian distribution from those histograms and we have chosen as threshold the value of log-selectivity where we find a valley in between the two peaks to have a stable classification.

In contrast with [16] we decided to fit a threshold for each experiment since in principle this quantity might change for each experimental realization, and as one can see from Fig. 3 this is the case.

It is evident from Figs. 2 and 3 that the bimodal Gaussian mixture distribution fits both the experimental data and the predictions very inaccurately. In fact, there is no reason why these quantities should follow such a distribution. In this case, the Gaussian mixture distribution is just the simplest thing one can do to select a threshold value for these quantities. In the following, it will be clear that this is not an issue because the robustness of our biophysical model isn’t affected by the choice of such a threshold as long as it is some sensible value between the two peaks. This is evident from the ROC curve analysis reported in "Results" section.

### Supervised binary classifier

To benchmark the classification performance of the energy-based model, we trained a neural network to perform viable/non-viable classification (binary classifier) in a supervised manner. The architecture of this classifier is identical to that of the energy-based model (refer to Sect. 2 in the Supplementary Material for details). Defining $q_{s,\text {viable}}$ and $q_{s,\text {non-viable}}$ as the two outputs of the Neural Network corresponding to the two states, the loss function can be expressed as:

10 $$\begin{aligned} d {\mathcal {C}}=\sum _s \left[ y_s \ln \frac{e^{q_{s,\text {viable}}}}{e^{q_{s,\,\text {viable}}}+e^{q_{s,\,\text {non-viable}}}} + (1-y_s) \ln \frac{e^{q_{s,\,\text {non-viable}}}}{e^{q_{s,\,\text {viable}}}+e^{q_{s,\,\text {non-viable}}}}\right] \end{aligned}$$

where $y_s$ is 1 if the sequence is labeled as viable and 0 otherwise.

## Results

To evaluate the model’s performance and its robustness, we conducted two comparisons. Firstly, we trained the models on sequences involving single mutations/insertions (those tested in *experiment-1*) and then used the trained models to infer the selectivity of sequences tested in *experiment-2*. Secondly, we trained the models on sequences from both *experiments 1 and 2* and employed them to predict the selectivity of sequences tested in *experiment-3*. Other train-test splits are possible, indeed. Anyway, we chose this procedure because experiments are substantially different one from another to represent a significant stress test to evaluate the generalization power of the models.

We assess the predictive capability of the models from two perspectives: their ability to forecast the selectivity of sequences (as defined in Eq.(1)) and their proficiency in classifying sequences as viable or non-viable (as detailed in "Binarizing the output of the energy-based model" section). Concerning the former, we calculated the Pearson correlation coefficient between the inferred log-selectivity from Eq. (1) and the empirical log-selectivity defined by Eq. (9). For the latter, we generated the confusion matrix of the binary classification, comparing the inferred viable/non-viable labels with the empirical ones assigned as described in "Data binarization" section. It’s important to note that when referring to the Pearson correlation, we specifically assess the $\log p_s$ inference, while discussions about accuracy and the confusion matrix pertain to the evaluation of binary classification.

**Fig. 4:**
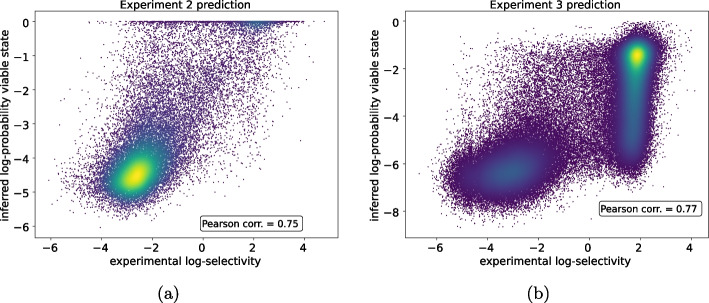
Scatter plot of empirical log-selectivities vs inferred log-probabilities of the viable state. **a** The model is trained on *experiment-1* then used to predict sequences from *experiment-2*. **b** The model is trained on *experiments 1 and 2* then used to predict sequences from *experiment-3*

In the first scenario, the neural network, trained on single mutations and insertions, achieves an accuracy of approximately 80% when tested in *experiment-2*. In contrast, the biophysical model attains an accuracy of about 88% on the same sequences. Despite the comparable binary classification accuracy, the biophysical model excels in providing precise information about the continuous ’selectivity’ values of the variants, providing reliable insights into the energy levels of the sequences. In the second scenario, predicting the selectivity of sequences in *experiment-3* proves more challenging as these sequences are in principle highly diverse from the ones in the training set. The neural network model achieves an accuracy of approximately 67%, whereas the biophysical model achieves an accuracy of about 81%. The drop in accuracy experienced by the neural network is substantial, whereas the biophysical model maintains high accuracy in this case. Furthermore, we highlight that the biophysical model also sustains a high correlation between empirical log-selectivities and inferred log-probabilities of the viable state. This indicates that the method is robust when the aim is to understand the relative ’fitness’ (the energy levels) of the sequences, as illustrated in Fig. 4a, b (with a Pearson coefficient of 0.75 in the first case and 0.77 in the second one).

**Fig. 5:**
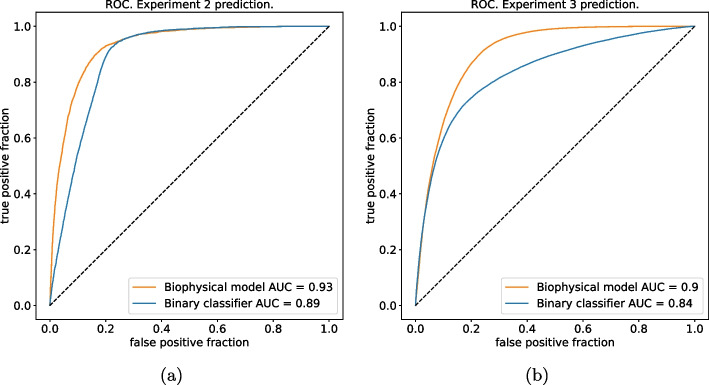
ROC curves of both models obtained by varying the classification threshold. **a** ROC curve of both models trained on data from *experiment-1* and used to predict sequences tested in *experiment-2*. **b** ROC curve of both models trained on data from *experiment-1* and *2* and used to predict sequences tested in *experiment-3*

We also explore the impact of varying the threshold value on the inferred $p_s$ by constructing a ROC curve. This analysis provides insights into the robustness of the classification concerning the choice of the threshold value. Similarly, a ROC curve is generated for the supervised Neural Network by varying the criterion used to classify sequences: instead of classifying the sequence as viable if $q_{s,\text {viable}} \ge q_{s,\text {non-viable}}$, we introduce a threshold $\tau$, classifying sequences as viable if $q_{s,\text {viable}} - q_{s,\text {non-viable}} \ge \tau$. The ROC curves for both models are illustrated in Fig. 5a, b for the two train/test combinations.

**Table 2:** Confusion matrices of the two models for each train/test combination

	Predicted	Predicted
	Viable	Non-viable
(a)		
Viable	4132	286
Non-viable	5130	16878
(b)		
Viable	3612	806
Non-viable	2474	19534
(c)		
Viable	33386	42924
Non-viable	3450	60874
(d)		
Viable	58964	17346
Non-viable	9271	55053

(a) Supervised binary classifier trained on *experiment-1* and tested on *experiment 2*. (b) Biophysical model trained on *experiment-1* and tested on *experiment 2*. (c) Supervised binary classifier trained on *experiment-1* and *experiment-2* and then tested on *experiment-3*. (d) Biophysical model trained on *experiment-1* and *experiment-2* and then tested on *experiment-3*

As one can see from Fig. 5a the area under the ROC is very high ($\approx 0.93$) when the model is trained on single mutations/insertions and used to test sequences from *experiment-2*. Figure 5b shows that when the model is trained on *experiments 1 and 2* and used to predict sequences from *experiment-3* the area under the ROC slightly decreases at $\approx 0.90$. Perhaps surprisingly, we observe from Fig. 5b that the performance of the binary classifier (blue lines) is marginally worse than the biophysical model (orange lines) in both experiments. The same trend, perhaps more clearly, is shown in Table 2 where we display the confusion matrices for the two models in each train/test combination. When training both models on *experiment-1* and testing the classification task on *experiment-2* (Table 2 first row), we obtain a relatively comparable performance: a true positive rate of  0.82 for the biophysical model, while  0.94 for the supervised binary classifier; a true negative rate of  0.89 for the biophysical model and of  0.77 for the binary classifier. Things change when we train on *experiment-1 and 2 * and we test on *experiment-3* (Table 2 second row). Here, with a true positive rate of  0.44 for the binary classifier, and  0.77 for the biophysical model, we double the figure while keeping a true negative rate roughly comparable (around 0.95 for the binary classifier vs. 0.86 for the biophysical model).

Evaluating the generalization capabilities of our model concerning the variant distance from the WT sequence is a compelling endeavor. This test holds particular interest due to the substantial heterogeneity in composition among the various training sets, as illustrated in Fig. 1. To elucidate further, *experiment-1* provides a relatively narrow view of the mutational landscape around the WT, encompassing only single inserts and mutations. Conversely, *experiment-2* and *experiment-3* explore a broader range of the mutational landscape, generating sequences at more substantial distances from the WT. Notably, *experiment-2* exhibits a higher concentration of sequences around the WT, with an exponential distribution tail extending up to a Levenshtein distance of 33. In contrast, the composition in *experiment-3* is more uniformly distributed across a similar range as explored by *experiment-2*. To evaluate the predictive capabilities of our models with respect to the distance from the WT sequence, we dissect the performance of the various models based on the distance of the test set sequences from the WT.

**Fig. 6:**
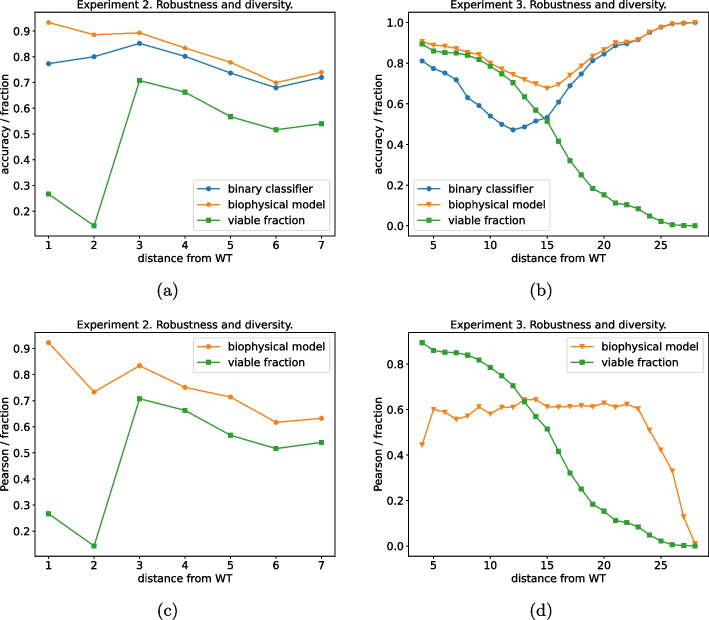
Robustness analysis with distance. The data set has been divided into slices according to their distance from wild-type and the models have been tested on those slices. The score for the two models is reported as a function of the distance of the test data. **a** The models are trained on *experiment-1* and tested on *experiment-2*. **b** The models are trained on *experiments 1 and 2* and tested on *experiment-3*. **c** The model is trained on *experiment-1* and tested on *experiment-2*. **d** The model is trained on *experiments 1 and 2* and tested on *experiment-3*

Again, we report the results for the model trained on *experiment 1* and tested on *experiment-2* (Fig.  6a, c) and for the model trained on *experiment-1,2* and tested on *experiment 3* (Fig. 6b, d). As a score, we use: the accuracy for the binary classifier, and the Pearson correlation coefficient between the model log-likelihood and the sequence empirical log-selectivity for the biophysical model.

From the green lines with square markers in Fig. 6 we immediately note how the viable fractions of the two test sets are remarkably different. Whereas *experiment-2* (Fig. 6a, c) shows a low fraction of viable variants at a small distance from the WT (Levenshtein distance lower equal than 2), this fraction increases around 0.6 at higher distances. The composition is radically different for *experiment 3* (Fig. 6b, d) where the viable fraction monotonously decreases as a function of the Levenshtein distance from the WT, from an initial value of around 0.9 to almost 0 for the most distant variants. When we compare the performance of the binary classification on the two datasets, for both models we observe that the unsupervised biophysical model systematically outperforms the supervised binary classifier although the margin is minimal when we consider as a test set *experiment-2* Fig. 6a, more pronounced for *experiment-3* Fig. 6b. In this second case, there is a tiny drop in performance at the intermediate distance from the WT sequence where viable and non-viable sequences are in almost equal shares, and arguably the classification task is more difficult.

Interestingly, when we assess the generalization performance of the biophysical model to predict the sequence log-selectivity when testing on *experiment-2* (Fig. 6c) we observe a mildly monotonously decreasing curve from very high correlation values (around 0.9 for the closest variants) down to a correlation of around 0.7 for the most distant variants. A different behavior is observed when testing on *experiment-3* (Fig. 6d), where, besides a decrease in correlation for the extreme values of the distance from the WT, a stable correlation of around 0.6 is observed.

We remark that there are different possibilities to test the robustness of our model, and looking at the prediction accuracy at increasing distance from WT is just one of the possibilities. We have chosen this criterion to be consistent with [16] and also because exploring functional variants as distant as possible from the WT sequence is the ideal objective of the biotechnological application. For completeness, an alternative metric is reported in Sect. 4 of the Supplementary Material.

**Fig. 7:**
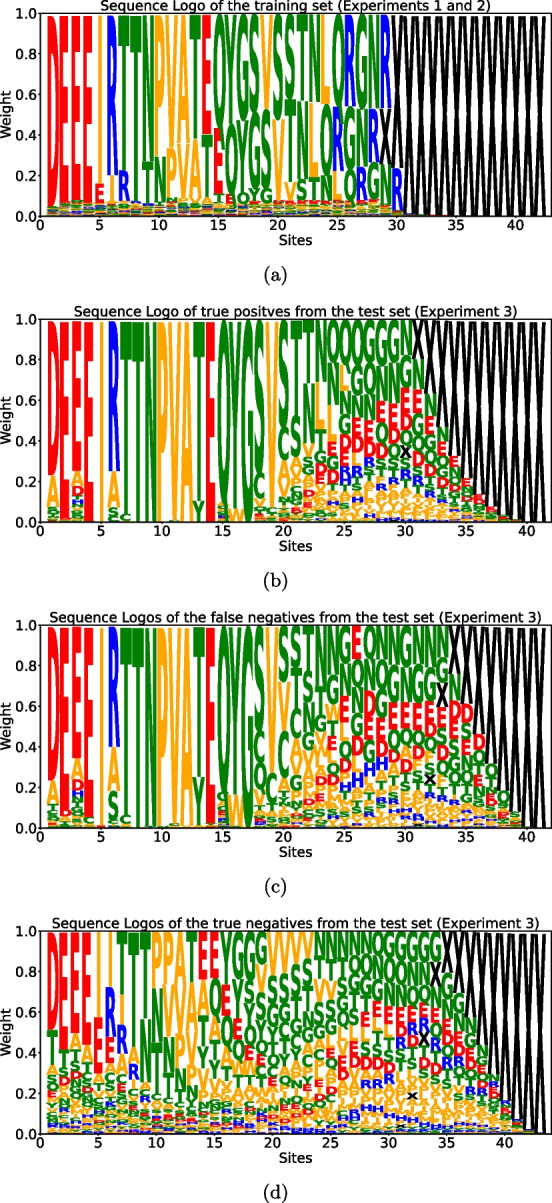
Sequence logos of some subsets of the tested sequences. The numbers on the *x* axis indicate the position along the sequence, starting from site 561 which corresponds to position 1. Those sites that are composed only of gaps within the specific subset have been cut out from the logo (for the details about the sequence encoding see Sect. 2 in the Supplementary Material). The height of each letter is proportional to the frequency of the corresponding amino acid in the specific sub-set of unique sequences. The color, instead, is related to the physico-chemical characteristics of the amino acids. In some of the panels, at the rightmost sites, the gaps are so much more frequent that it is difficult to see the other small letters appearing. **a** Sequence logo of sequences of *experiment-1* and *experiment-2*. **b** Sequence logo of sequences from *experiment-3* that are correctly classified as viable. **c** Sequence logo of sequences from *experiment-3* that are classified as non-viable but have been tested as viable. **d** Sequence logo of sequences from *experiment-3* that are correctly classified as non-viable

Despite the overall quantitatively and qualitatively good generalization properties of our models shown by the above-mentioned results, a prominent challenge that our biophysical model still grapples with is the task of learning discernible patterns from the viable sequences examined in *experiment-3*. As shown from Fig. 4b, it’s apparent that the model tends to produce false negatives. A plausible explanation for this observation becomes evident when we compare the patterns of true positives and false negatives with those of genuinely non-viable sequences. Figures 7b, c exhibit a strikingly similar composition, and we aspire for our model to correctly categorize all such sequences as viable, given their apparent similarities. However, when we contrast these patterns with those in Fig. 7a, which represents the sequences used for training, we can discern that the model has been exposed to significantly different patterns than those we require it to recognize, particularly in the rightmost portion of the sequences. On the other hand, the model generally correctly classifies non-viable sequences because they manifest entirely distinct patterns, particularly in the leftmost section of the sequence, as illustrated in Fig. 7d.

As we anticipated in "Binarizing the output of the energy-based model" section, there isn’t any prescribed way to assign a threshold for the binary classification using the inferred energies $E_s$ (or equivalently the selectivities $p_s$), the best way to do it might depend on the data and the task we want to accomplish. We will now make a comparison between the binary classification performed using the threshold value obtained by fitting a bimodal Gaussian distribution for the inferred energies $E_s$ and a threshold value that is obtained in a standard way if we knew the empirical labels of the sequences: by maximizing what is known as g-means score. To do so, first, we use the inferred parameters to sort the variants according to their value of selectivity $p_s$. Let us call this ordering permutation $\pi _s \in \{1,\ldots , S\}$ such that $\pi _s \le \pi _\sigma$ if and only if $p_s \ge p_\sigma$. Then for each $s \in S$, we compute the fraction of empirical positive and negative sequences in the set $\{s' | \pi _{s'} \le s\}$ according to their empirical $\theta$ value. This corresponds to setting the threshold equal to $p_s$ such that *s* is the first sample (according to the permutation $\pi$) to be considered as viable. Then, for each value of the threshold, we compute the *g-means score* defined as:

11 $$\begin{aligned} \text {g-means}(p_s) = \sqrt{\text {true positive rate}(p_s) \times (1-\text {false positive rate}(p_s))} \end{aligned}$$

**Fig. 8:**
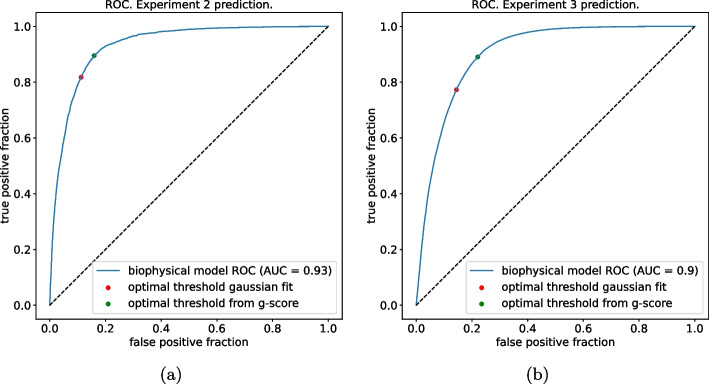
The blue curves are the same ROCs showed in Fig. 5a, b just for the biophysical model in this case, on top of them are plotted the two working points of the binary classification, both in case in which the threshold is fitted by the inferred energies distribution (unsupervised) and in the case in which the threshold is obtained using the labels of the test sequences (biased). **a** The model is trained on *experiment-1* and tested on *experiment-2*. **b** The model is trained on *experiments 1 and 2* and tested on *experiment-3*

Then, we look for $s^*=\text {argmax}_s \{\text {g-means}(p_s)\}$, thereby, the corresponding value of $p_{s^*}$ sets the optimal threshold value for the binary classification. Figure 8 shows the points on the ROC corresponding to the thresholds estimated in the two ways; it is evident that in this case, a bimodal fit on the energies distribution finds a threshold value that has a very similar performance to the one that uses a threshold that we would find if we used the hidden information from the test set.

## Conclusions

We introduce a novel biophysically-inspired model designed for the prediction of the viability of Adeno-associated virus (AAV) capsids. Leveraging information from Deep Mutational Scanning (DMS) experiments, as elaborated in [16], the model learns the phenotypes of genetic variants within the CAP region of the AAV2 capsid protein.

To assess the model’s efficacy, three datasets were employed, encompassing diverse variant libraries engineered to explore different facets of the mutational landscape. Our exploration of the biophysical model’s generalization capabilities focused on two primary objectives: (i) delivering quantitative forecasts for the log-selectivity of variants and (ii) deploying it as a binary classifier, categorizing sequences into viable and non-viable classes.

The model employs a probabilistic framework describing three distinct phases of the experiment: amplification, selection, and sampling. This framework defines the likelihood of observing a variant as the selection process proceeds. Crucial to this is the parametrization of the sequence-to-fitness map, linking a variant’s genotype to its ability to form a capsid, i.e., viability. The sequence-to-fitness map is parametrized using a Neural Network (NN) trained to maximize the likelihood of the entire experiment. Notably, the training follows an unsupervised approach, excluding the viability label as part of the training data. Post-training, the model can classify sequences based on the sequence-to-fitness map.

To benchmark our model’s ability, we compare it to a binary classifier distinguishing between viable and non-viable outcomes. This classifier employs a dense Neural Network with the same architectural framework as the biophysical model but undergoes supervised training, enhancing its precision in classifying sequences.

An outstanding feature of the DMS library in [16] is its inclusion of insertions, not just mutations, in the wild-type sequence. This results in libraries, depicted in Fig.  7, containing fragments of variable lengths. To effectively handle non-aligned sequences, both models integrate some convolutional layers that process the amino-acid sequence input before feeding it to the dense ones.

In conclusion, our results support the introduction of a biophysical model that provides a robust and interpretable computational framework to model the intricate mutational landscape characteristic of DMS experiments. The model proves useful in predicting and selecting viable AAV2 capsids, demonstrating high generalization power to predict out-of-sample test variants with considerable sequence diversity from the training set.

The model could be employed to generate novel variants predicted to produce viable capsids with high sequence diversity compared to the wildtype. In our future outlook, we envision leveraging the physics-informed machine learning approach to generate variant sequences, optimizing critical molecular properties like viability. Furthermore, this methodology holds significant potential for integration with other models aimed at optimizing diverse properties, including tropism. This presents a compelling pathway for engineering viral capsids tailored for use as viral carriers.

Furthermore, while the constraints to engineering a suitable vector and maintaining a live viral reservoir are evidently different, the design of AAV2 vectors is still likely to be informed/influenced by what we know about the natural evolution of the virus. A possible further development in this line is twofold. First, one could compare using some dimensionality reduction based analysis a la PCA how naturally evolving sequences compare with artificial ones. Second, and perhaps more interesting, would be to add to the training dataset, the set of natural homologs present in the databases (e.g. PFAM) that display mutations to the reference WT in the segment artificially mutated to see if and how our model predictions improve.

### Supplementary Information

Supplementary file.

## Data Availability

A GitHub repository, containing the code necessary to implement and run our method, can be found at this link. This repository includes Jupyter Notebooks and detailed tutorials to facilitate the replication of the results presented in our manuscript.
